# Remote and adjacent psychological predictors of early-adulthood resilience: Role of early-life trauma, extraversion, life-events, depression, and social-support

**DOI:** 10.1371/journal.pone.0251859

**Published:** 2021-06-24

**Authors:** Sitong Shen, Zhaohua Chen, Xuemei Qin, Mengjia Zhang, Qin Dai

**Affiliations:** 1 Department of Nursing Psychology, Army Medical University, Chongqing, China; 2 Department of Psychology, Army Medical University, Chongqing, China; 3 Institute for Brain and Psychological Sciences, Sichuan Normal University, Chengdu, China; Guangzhou University of Chinese Medicine, CHINA

## Abstract

Resilience is important for people to maintain mental health after negative life-events. However, its longitudinal psychological and social predictors are poorly revealed. Based on the ecological system theory model, the current study aimed to determine the longitudinal temporal mechanism underlying the development of early-adulthood resilience using long-term (early-life trauma and personality), medium-term and short-term (life-events, social support, and depression) psychosocial predictors. A total of 505 university students were recruited at baseline (T1), 433 of whom took part in a three-year longitudinal investigation (T2). The results showed that at T1 and T2, the resilience scores of individuals were identically high (72.98 and 73.21, respectively). Pearson correlation analysis showed that early-adulthood resilience was negatively correlated with early-life trauma, psychoticism and neuroticism, depression, ad life-events, and positively correlated with extraversion, social-support, and resilience. Regression and structural equation models showed that extraversion had a direct positive effect on T1 resilience through the mediation of T1 life-events, depression, and social-support, while childhood emotional neglect (EN) had indirect negative effect and extraversion had direct positive effect on T2 resilience through the mediation of T1 resilience, and T2 depression and social-support. In conclusion, this study is among the first to reveal the longitudinal temporal process of the development of early-adulthood resilience using remote and adjacent psychosocial predictors. The findings confirm that childhood EN and extraversion have a remote impact on early-adulthood resilience through recent and current depression and social-support. Our results imply that early-life trauma does not hinder the development of early-adulthood resilience in a linear trend.

## Introduction

Resilience, or hardiness, is a capability or character trait that helps individual overcome the ups and downs of daily life [1], and further assist people to maintain an emotional balance after a traumatic experience [2]. Previous studies have confirmed that resilience is highly effective in improving lifetime mental health after traumatic life-events [2, 3].

Early-adulthood (18–25 years old, including students of university age) is a critical developmental transition [4] but also a particularly vulnerable period to mental health illness [5], which might be potentially important to the foster of resilience during this period. Young adulthood is therefore an important stage to the development of resilience, which mediates health-related quality of life [6]. However, little attention has been paid to resilience during early-adulthood in existing literature, resulting in a limited empirical guidance for and necessitating a better understanding of its development.

Many factors contribute to the development of resilience [7]. According to the ecological system theory [8], the risk and protective factors of personal mental health can be assessed from different ecological levels: personal (e.g., personality), family (e.g., early-life trauma and parental style) or social variables (e.g., recent life-events and social support). Thus, the development of resilience may be predicted by both personal psychological traits and family or social factors, which emphasizes the collective effects of psychosocial contributors.

As a long-term family or social predictor, psychologists assumed that maltreatment during early-life (mainly before 16 years of age) [9, 10] diminished emotional processing and social competence in children and adolescents [11], which was commonly known as a destructive factor for personal mental health problems such as depression and post-traumatic stress disorder [10, 12]. Indeed, both cross-sectional and longitudinal studies have shown that individuals who experience early-life trauma are at high risk for lifetime mental health problems such as depression, including depression in the early-adulthood [13–16]. Although the effect of early-life trauma on lifetime psychological disturbance has been well documented, little is known about the long-term predictive effect of early-life trauma on adult resilience. Some studies linked early-life trauma to personal resilience [2, 17]. Other studies showed that positive early-life experiences helped to instill resilience during early-adulthood [18], and resilience acted as a protective mechanism against the effect of early-life trauma on psychological disorders among young adult [19]. However, the long-term predictive effect of early-life trauma on early-adult resilience remains largely unknown. This knowledge may be important for future family and school education in fostering higher resilience in children by mitigating the harm stemming from early-life experiences.

The impact of exogenous early-life trauma on early-adulthood resilience might be mediated by endogenous factors such as personality, which is relatively stable even during the emerging adulthood [20]. A study found that high extraversion predicted resilience [21]. Extraversion has been emphasized by researchers as positively influencing mental health, especially resilience [22, 23]. Similarly, extraversion plays a mediating role in the relationship between early-life hardship and adult resilience [17]. In young adult, it has been confirmed that resilience is positively related to extraversion [24]. These results suggested that the endogenous extraversion personality trait foster resilience; however, whether it mediates the effect of early-life trauma on early-adulthood resilience remains unclear.

Importantly, in an earlier famous longitudinal study, Werner [25] found that children with poor resilience would get a promotion in resilience later when they had learn more about coping with adversity, and suggested a potential short-term mediation mechanism between early-life trauma and early-adulthood resilience, which contains a risk and resilience framework [26, 27], such as depression (risk factor) [28], life-events (risk factor) [29], and social support (protective factor) [30]. Notably, study has shown that depression in early-adulthood is associated with early-life maltreatment and low resilience [31]. Depression mediates the relationship between early-life trauma and long-term functional outcomes [28]. In addition, depression has a passive effect on resilience [32] and has been found to mediate (15.6%) the influence of resilience on addictive behavior [33]. Besides depression, life-events have been indicated a role in resilience. Study reported that the effects of exposure to poverty on hippocampal development were mediated by stressful life-events [34]. Similarly, Ganzel [35] found that healthy adults experiencing stressful life-events had reduced gray matter volume in the amygdala and left anterior hippocampus, which are regions that regulate stress adaptation and largely influence resilience. More specifically, severe life-events such as parental cancer are associated with lower resilience [36]. However, an animal study showed that predictable chronic mild stress during adolescence positively affected brain functioning and enhanced resilience in adults [29]. Moreover, social support also has an effect on resilience. Social support when facing stressful events improves quality of life [37], and mediates the relationship between early-life trauma and allostatic load across the life span [30]. Likewise, friendship support predicts both immediate and later resilience [38, 39]. Together, the literature suggests that depression, life-events, and social-support have short-term impacts on resilience, however, the impact of these factors on early-adult resilience has not been confirmed.

One-point deserves to point out is that, the statuses of dynamic life-events and social supports vary across different time-points, influencing resilience differently [40], and cannot be captured dynamically by a one-time observation. However, the statuses of dynamic factors (i.e., life-events) has often been observed and reported at a single point in time instead of at multiple points [41]. The study warranted a multi-time point observation of dynamic psychosocial-status (i.e., depression, life-events, and social support) based on the ecological system theory model, which may provide insight into the temporal mechanism underlying the development of early-adulthood resilience, using long-term and short-term predictors.

In conclusion, based on the ecological system theory model, the current study aimed to identify the temporal process underlying the development of early-adulthood resilience using long-term (early-life trauma and personality), medium-term and short-term (recent and current life-events, social support, and depression) psychosocial predictors. Our hypotheses were as follows: (1) long-term psychosocial vulnerabilities, i.e., early-life trauma and personality (i.e., extraversion) may have remote effects on early-adulthood resilience; (2) recent life-events, social support, and depression may play a medium-term mediating role in the relationship between long-term psychosocial vulnerability and early-adulthood resilience; and (3) current life-events, social support, and depression may play a short-term mediating role in the relationship between long-term psychosocial vulnerability and early-adulthood resilience.

## Materials and methods

### Participants

A total of 505 university freshmen in Chongqing, China, were recruited for this three-year longitudinal investigation in 2016. The participants were investigated with the Beck depression inventory-II (BDI-II), the Childhood Trauma Questionnaire (CTQ), the Eysenck personality questionnaire (EPQ), the Social-Support Rating Scale (SSRS), the Connor-Davidson Resilience Scale (CD-RISC), and the Adolescent Self-Rating Life-Events Checklist (ASLEC). To reduce the interference effect of severe physical illness and psychosis, participants were further screened by psychologists to exclude current or previous substance/alcohol misuse or Axis I psychiatric disorders based on the Diagnostic and Statistical Manual of Mental Disorders (DSM-IV-TR) [42]. Exclusion criteria included having a learning disability, having sustained severe physical trauma, receiving psychiatric medication, having received a diagnosis of general anxiety disorder, bipolar disorder, lifetime or current psychotic symptoms, and substance (including alcohol) dependence or abuse within the past six months. Three students were excluded (one with severe head trauma, one with general anxiety disorder, and one with current psychotic symptoms), and 505 students completed the baseline investigation. Students were further invited to take part in a three-year longitudinal investigation from 2016 to 2018, 433 of whom completed the three-year longitudinal survey (M/F: 389/44; aged between 17 and 22 years, 18.94±1.44). As the participants were from a military medical university, and they all majored in clinical medicine, there were more male participants than female participants.

### Instruments

To evaluate the levels of resilience, childhood trauma, personality, social support, life-events, and depression in participants, mature questionnaires with good validity were selected, which guaranteed the investigation power of this study.

To assess resilience, the Chinese version of Connor-Davidson Resilience Scale (CD-RISC) was used, which comprises 25 items. The internal consistency reliability coefficients of its three factors (tenacity, strength, and optimism) have been reported to be 0.88, 0.80, and 0.60, respectively [43].

To collect information about childhood trauma (before 16 years old age), the Childhood Trauma Questionnaire (CTQ) was employed [44]. The short version of the CTQ comprises 28 items that assess emotional abuse (EA), physical abuse (PA), sexual abuse (SA), emotional neglect (EN), and physical neglect (PN). The total CTQ score considers the severity of multiple forms of abuse and neglect. The internal consistency reliability coefficients of the original version range from 0.61 (PN) to 0.95 (SA) [44]. In the current study, the CTQ scores ranged from 28 to 88, the mean CTQ score was 37.55, and 189 students scored higher than the mean score.

To assess personality, the Eysenck personality questionnaire (EPQ, 1983 version, 88 questions) was used. The reliability of this instrument has been tested in China, yielding a Cronbach’s alpha coefficient of 0.86 [45]. The total score is the sum of all “yes” answers, and higher total scores indicate that the given personality trait is more characteristic of the responder.

To assess social-support, the Chinese version Social-Support Rating Scale (SSRS) was used, which was designed by Shuiyuan Xiao [46, 47], and had 10 items in total. The SSRS comprises three dimensions: objective support (three items, e.g., in the past, when you were in an emergency, the sources of comfort and care were), subjective support (four items, e.g., how many close and supportive friends have you had during the past year), and the utility of support (three items, e.g., when you were in trouble, your help mode was). Higher scores indicate higher levels of support from family and society. The Cronbach’s alpha coefficient was 0.92.

To collect life-stress information, the Adolescent Self-Rating Life-events Checklist (ASLEC) [48, 49] was used, which was designed by Liu in 1997 [50]. This scale is a retrospective self-report questionnaire that includes 27 items measuring the frequency and intensity of negative life-events. The scale has good psychometric properties, with a Cronbach’s alpha coefficient of 0. 85.

To assess depression levels, Beck depression inventory (BDI-II) was used. The BDI-II is a self-report scale with 21-items corresponding to the severity of depressive symptoms [51, 52]. Scores equal to or lower than 4 represent no depression; scores between 5 and 13 represent mild depression; scores between 14 and 20 represent moderate depression; scores equal to or above 21 represent severe depression.

### Procedure

The study protocol was approved by the Human Research Ethics Committee of the Army Medical University. University freshmen were invited to participate in this investigation in both verbal and written format in 2016. Responders who agreed to participate signed the written informed consent form of this research. Subsequently, the survey was carried out in quiet classrooms. Variables observed in 2016 (T1) were: personality, childhood trauma, depression, life-events, social-support, and resilience. Variables observed in 2018 (T2) were: depression, life-events, social-support, and resilience. The subjects received course credit for their participation after the third year’s investigation.

### Statistics

To address our hypotheses, the following analyses were carried out. A paired t-test was performed to compare the resilience status of the subjects between T1 and T2, Cohen’s d was calculated for effect size. Pearson correlation analyses were computed to examine the correlations among childhood trauma, personality, resilience, life-events, social-support, and depression, Bonferroni’s correction was carried out for multiple correlations (p < 0.001). To screen for significant data-based predictors, and provide more data-based support for the hypotheses, a hierarchical linear regression analysis was conducted to examine the impact of demographic variables (age and gender, first layer), longstanding personality and early-life trauma (second layer), T1 psychosocial status (third layer) and T2 psychosocial status (fourth layer) on T1 and T2 resilience, which effect was evaluated according to Cohen’s guidelines [53]. Specifically, demographic variables (age and gender) were included and controlled during the regression analysis, since that the college students in this study were same in college educational level and unmarried status, and different in age and gender. Structural equation modeling (Due to SEM model, alpha adjustments were not made) was carried out with AMOS 24.0 to test the direct and mediating effects of predictors on T1 and T2 resilience [54], and potential contributors included in the equations were the significant predictors in the regression analysis. Bootstrap tests (2000 repeated samples and 95% confidence intervals) were used to test the significance of the mediating effect, with 95% CIs not containing 0 indicating a significant mediating effect.

## Results

### Basic information about the early-adulthood resilience (Table 1)

In total, 433 students completed the four-year longitudinal study involving two surveys (T1 conducted in 2016, T2 conducted in 2018). The resilience level of participants at T1 and T2 were 72.98 and 73.21, respectively, and the paired t-test showed that they did not differ significantly, t (432) = -0.30, p = 0.764, Cohen’s d = -0.03.

**Table 1:** Basic information about the early-adulthood resilience.

	Gender		Age	Resilience			
	Male/Female (N)			Tenacity	Strength	Optimism	Total scores
T1	389/44	Mean	18.94	36.45	25.07	11.46	72.98
		S.D.	1.44	8.55	4.76	2.65	14.80
T2		Mean		36.87	24.81	11.52	73.21
		S.D.		8.23	4.78	2.55	14.55
**t**				-0.958	0.985	-0.461	-0.30
Cohen’s d				-0.09	0.09	-0.04	-0.03

Note: T1 = Year 2016. T2 = Year 2018.

### Correlation between T1 and T2 resilience and psychosocial variables (Table 2)

With the variables of personality and early-life trauma, T1 and T2 psychosocial status (resilience, depression, social support, and life-events), Pearson correlation analysis was conducted. The results indicated that early-adulthood resilience at T1 and T2 was negatively correlated with EA, EN, PN, psychoticism and neuroticism of EPQ, depression and life-events of T1 and T2 (r = -0.17 ~ -0.52). Additionally, early adulthood resilience was positively correlated with extraversion and social support at T1 and T2 (r = 0.19–0.55). T1 resilience and T2 resilience was positively correlated (r = 0.39).

**Table 2:** Pearson correlation between resilience and psychosocial variables.

												T1					T2				
		EA	PA	SA	EN	PN	CTQ	P	E	N	L	ASLEC F	ASLEC Q	Resilience	SS	BDI	ASLEC F	ASLEC Q	Resilience	SS	BDI
**Long-term vulnerability**	EA	1.00																			
	PA	0.54*	1.00																		
	SA	0.41*	0.53*	1.00																	
	EN	0.35*	0.33*	0.20*	1.00																
	PN	0.21*	0.23*	0.27*	0.54*	1.00															
	CTQ	0.59*	0.60*	0.49*	0.91*	0.68*	1.00														
	P	0.09	0.08	0.04	0.12	0.08	0.13	1.00													
	E	-0.02	0.02	0.01	-0.20*	-0.16	-0.16	-0.21*	1.00												
	N	0.11	0.10	0.04	0.21*	0.13	0.20*	0.41*	-0.31*	1.00											
	L	-0.07	-0.05	0.05	-0.08	-0.09	-0.08	-0.36*	0.15	-0.48*	1.00										
**T1**	ASLEC F	0.19*	0.17*	0.21*	0.10	0.14	0.19*	0.16	-0.12	0.29*	-0.11	1.00									
	ASLEC Q	0.17*	0.20*	0.16	0.14	0.13	0.20*	0.19*	-0.11	0.32*	-0.15	0.77*	1.00								
	Resilience	-0.04	-0.04	0.02	-0.23*	-0.18*	-0.20*	-0.12	0.34*	-0.22*	0.12	-0.35*	-0.36*	1.00							
	SS	-0.09	-0.07	0.02	-0.26*	-0.12	-0.22*	-0.17*	0.31*	-0.20*	0.09	-0.18*	-0.16	0.55*	1.00						
	BDI	0.11	0.13	0.04	0.23*	0.18*	0.23*	0.19*	-0.19*	0.32*	-0.16	0.46*	0.49*	-0.52*	-0.42*	1.00					
**T2**	ASLEC F	0.26*	0.22*	0.20*	0.08	0.06	0.17*	0.09	-0.09	0.14	-0.06	0.32*	0.31*	-0.12	-0.12	0.22*	1.00				
	ASLEC Q	0.27*	0.20*	0.16	0.14	0.06	0.21*	0.09	-0.12	0.17	-0.03	0.22*	0.29*	-0.15	-0.12	0.25*	0.82*	1.00			
	Resilience	-0.15	0.00	-0.03	-0.28*	-0.17*	-0.25*	-0.11	0.30*	-0.20*	0.08	-0.22*	-0.21*	0.39*	0.33*	-0.30*	-0.29*	-0.38*	1.00		
	SS	-0.11	-0.05	-0.01	-0.27*	-0.15	-0.24*	-0.17*	0.23*	-0.17	0.12	-0.06	-0.07	0.19*	0.40*	-0.15	-0.14	-0.23*	0.44*	1.00	
	BDI	0.28*	0.10	0.04	0.21*	0.10	0.23*	0.13	-0.12	0.27*	-0.13	0.21*	0.29*	-0.20*	-0.20*	0.42*	0.38*	0.55*	-0.52*	-0.29*	1.00

Note: * Bonferroni’s correction for multiple correlations: p < 0.001. T1 = Year 2016. T2 = Year 2018. EA = Emotional abuse. PA = Physical abuse. SA = Sexual abuse. EN = Emotional neglect. PN = Physical neglect. CTQ = Child trauma questionnaire. P = Psychoticism/Socialisation. E = Extraversion/Introversion. N = Neuroticism/Stability. L = Lie/Social Desirability. ASLEC = Adolescent Self-Rating Life-events Checklist. F = Frequency. Q = Quantity. SS = Social-support. BDI = Beck depression inventory.

### Hierarchical linear regression for T1 and T2 resilience (Tables 3 and 4)

To observe the predictors of T1 resilience, demographic variables (age and gender, first layer), early-life trauma and personality (second layer) and T1 psychosocial status (third layer) were included in a hierarchical linear regression. The results showed that extraversion of EPQ, T1 life-events, depression and social-support significantly predicted T1 resilience, accounting for 44.3% of the variance in T1 resilience. This qualified as a large effect according to Cohen’s guidelines [53]. Demographic variables, longstanding variables, and T1 psychosocial variables accounted for 0.3%, 14.4%, and 29.6% of the variance in T1 resilience, in which T1 depression was the most significant negative predictor, and T1 social-support was the most significant positive predictor.

**Table 3:** Hierarchical linear regression for T1 resilience.

		Model 1				Model 2				Model 3			
		B	Beta	t	p	B	Beta	t	p	B	Beta	t	p
**Demographic**	**Gender**	-2.14	-0.04	-0.88	0.38	-1.07	-0.02	-0.47	0.64	-0.11	0.00	-0.06	0.95
	**Age**	0.71	0.07	1.37	0.17	1.01	0.10	2.02	**0.04**	0.40	0.04	0.98	0.33
**Longstanding**	**EA**					0.32	0.03	0.59	0.56	0.37	0.04	0.82	0.41
	**PA**					-0.31	-0.03	-0.56	0.58	0.23	0.02	0.52	0.60
	**SA**					0.61	0.05	0.94	0.35	0.36	0.03	0.67	0.50
	**EN**					-0.30	-0.12	-2.14	**0.03**	-0.05	-0.02	-0.45	0.65
	**PN**					-0.72	-0.10	-1.77	0.08	-0.53	-0.07	-1.63	0.10
	**P**					0.00	0.00	0.03	0.97	0.11	0.06	1.51	0.13
	**E**					0.41	0.27	5.48	**<0.01**	0.24	0.16	3.89	**<0.01**
	**N**					-0.14	-0.10	-1.76	0.08	0.05	0.04	0.78	0.44
	**L**					0.00	0.00	-0.04	0.97	0.03	0.02	0.52	0.60
**T1**	**BDI**									-0.64	-0.24	-5.04	**<0.01**
	**ASLEC F**									-0.13	-0.05	-0.81	0.42
	**ASLEC Q**									-0.35	-0.15	-2.45	**0.01**
	**SS**									0.80	0.38	8.69	**<0.01**
**R**^2^		0.007				0.17				0.463			
**Standard R**^2^		0.003				0.147				0.443			
**F**		1.557				7.486				22.97			
**P**		0.21				<0.001				<0.001			

Note: T1 = Year 2016. EA = Emotional abuse. PA = Physical abuse. SA = Sexual abuse. EN = Emotional neglect. PN = Physical neglect. CTQ = Child trauma questionnaire. P = Psychoticism/Socialisation. E = Extraversion/Introversion. N = Neuroticism/Stability. L = Lie/Social Desirability. ASLEC = Adolescent Self-Rating Life-events Checklist. F = Frequency. Q = Quantity. SS = Social-support. BDI = Beck depression inventory.

**Table 4:** Hierarchical linear regression for T2 resilience.

		Model 1				Model 2				Model 3				Model 4			
		B	Beta	t	p	B	Beta	t	p	B	Beta	t	p	B	Beta	t	p
**Demographic**	**Gender**	-3.14	-0.07	-1.33	0.19	-2.60	-0.05	-1.18	0.24	-1.99	-0.04	-0.94	0.35	0.30	0.01	0.16	0.87
	**Age**	0.67	0.07	1.32	0.19	0.82	0.08	1.71	0.09	0.50	0.05	1.07	0.29	0.08	0.01	0.21	0.84
**Longstanding**	**EA**					-1.17	-0.12	-2.19	**0.03**	-1.21	-0.13	-2.37	**0.02**	0.03	0.00	0.06	0.96
	**PA**					1.26	0.13	2.38	**0.02**	1.46	0.15	2.86	**<0.01**	1.05	0.11	2.40	**0.02**
	**SA**					0.10	0.01	0.16	0.87	-0.03	0.00	-0.06	0.95	-0.28	-0.02	-0.54	0.59
	**EN**					-0.54	-0.23	-3.94	**<0.01**	-0.43	-0.18	-3.21	**<0.01**	-0.27	-0.11	-2.29	**0.02**
	**PN**					-0.26	-0.04	-0.67	0.51	-0.06	-0.01	-0.17	0.87	-0.11	-0.02	-0.35	0.73
	**P**					-0.01	-0.01	-0.13	0.89	0.01	0.01	0.17	0.87	0.03	0.02	0.43	0.67
	**E**					0.32	0.22	4.45	**<0.01**	0.21	0.14	2.90	**<0.01**	0.17	0.11	2.74	**0.01**
	**N**					-0.12	-0.09	-1.65	0.10	-0.04	-0.03	-0.56	0.57	0.05	0.03	0.70	0.48
	**L**					-0.05	-0.04	-0.68	0.50	-0.04	-0.03	-0.57	0.57	-0.02	-0.02	-0.39	0.70
**T1**	**BDI**									-0.22	-0.09	-1.50	0.14	0.10	0.04	0.72	0.47
	**ASLEC F**									-0.08	-0.03	-0.40	0.69	-0.21	-0.08	-1.26	0.21
	**ASLEC Q**									-0.08	-0.03	-0.47	0.64	0.12	0.05	0.84	0.40
	**SS**									0.15	0.07	1.32	0.19	-0.06	-0.03	-0.57	0.57
	**Resilience**									0.18	0.19	3.17	**<0.01**	0.23	0.23	4.59	**<0.01**
**T2**	**BDI**													-0.85	-0.37	-7.24	**<0.01**
	**ASLEC F**													-0.27	-0.06	-0.90	0.37
	**ASLEC Q**													-0.09	-0.04	-0.55	0.59
	**SS**													0.54	0.25	5.78	**<0.01**
**R**^2^		0.01				0.186				0.2615				0.475			
**Standard R**^2^		0.005				0.163				0.2365				0.448			
**F**		2.083				8.346				8.973				17.803			
**P**		0.126				<0.001				<0.001				<0.001			

Note: T1 = Year 2016. T2 = Year 2018.

To observe the predictive effects of the variables on T2 resilience, demographic variables (age and gender, first layer), early-life trauma and personality (second layer), T1 psychosocial status (third layer) and T2 psychosocial status (fourth layer), were included in a hierarchical linear regression. The results showed that extraversion of EPQ, childhood PN and EN, T1 resilience, T2 depression and social support significantly predicted T2 resilience, accounting for 44.8% of the variance of T2 resilience. This qualified as a large effect according to Cohen’s guidelines [53]. Demographic variables, longstanding variables, T1 and T2 psychosocial variables accounted for 0.5%, 15.8%, 7.25%, and 21.15% of the variance in T2 resilience, in which T2 depression was the most significant risk predictor, and T2 social-support was the most significant protective predictor.

### Structural equation model for T1 and T2 resilience (Figs 1 and 2 and Table 5)

**Fig 1:**
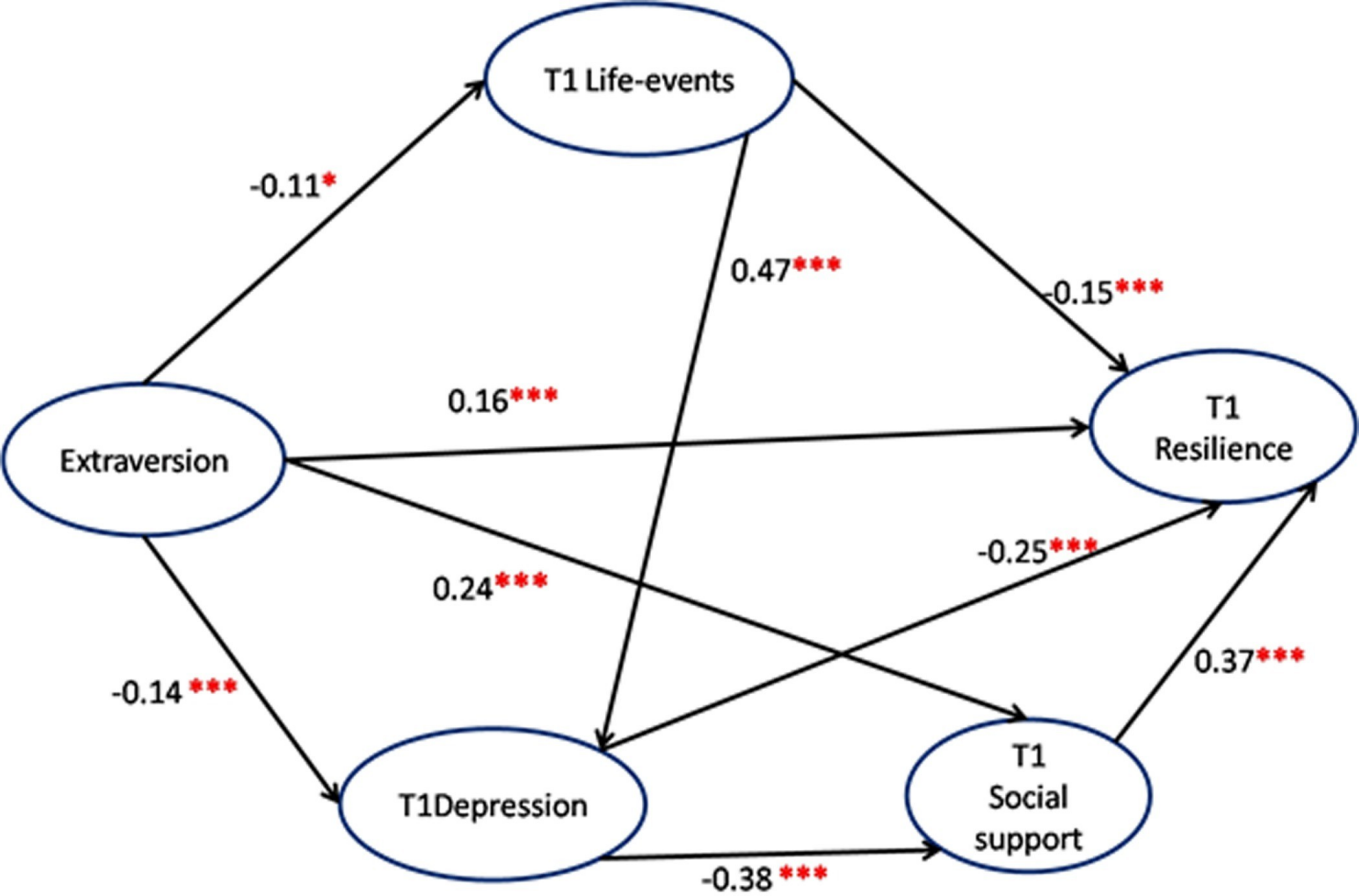
Structural equation model for T1 resilience. Note: *** P < 0.001. ** P < 0.01. T1 = Year 2016. Key information of model: CFI = 0.999, IFI = 0.999, NFI = 0.997, RFI = 0.966, RMSEA = 0.04, CMIN/DF = 1.698.

Based on the regression results, we developed a hypothesis-driven (Hypothesis 1: long-term psychosocial vulnerabilities, i.e., early-life trauma and personality (i.e., extraversion) may have remote effects on early-adulthood resilience and Hypothesis 2: Recent life-events, social support, and depression may play a medium-term mediating role in the relationship between long-term psychosocial vulnerability and early-adulthood resilience) structural equation model with the variables of extraversion of personality, T1 life-events, depression, and social support. Potential constructs between variables were based on the literature. Non-significant pathways were removed from the final model. The model fit test showed the fit index, CFI = 0.999, IFI = 0.999, NFI = 0.997, RFI = 0.966, RMSEA = 0.04, CMIN/DF = 1.698, which indicated that the model fit was good. Fig 1 shows that extraversion had direct (0.16, p < 0.01) and indirect effect (0.176, p = 0.001) on T1 resilience, the corresponding 95% CI (0.122, 0.238) without 0, indicating significant mediating effects between extraversion and T1 resilience. Moreover, Table 5 shows that there were direct and partial mediating effects between T1 life-events (direct effect = -0.15, p <0.001; indirect effect = -0.186 p = 0.002) and T1 depression (direct effect = -0.25, p < 0.001; indirect effect = -0.138, p = 0.001) on T1 resilience, and only a direct effect of T1 social-support (direct effect = 0.37, p <0.001) on T1 resilience. In which, extraversion and T1 social-support (strongest protector) had positive prediction on T1 resilience, while T1 depression (strongest risk) and life-events had negative predictions on T1 resilience.

**Fig 2:**
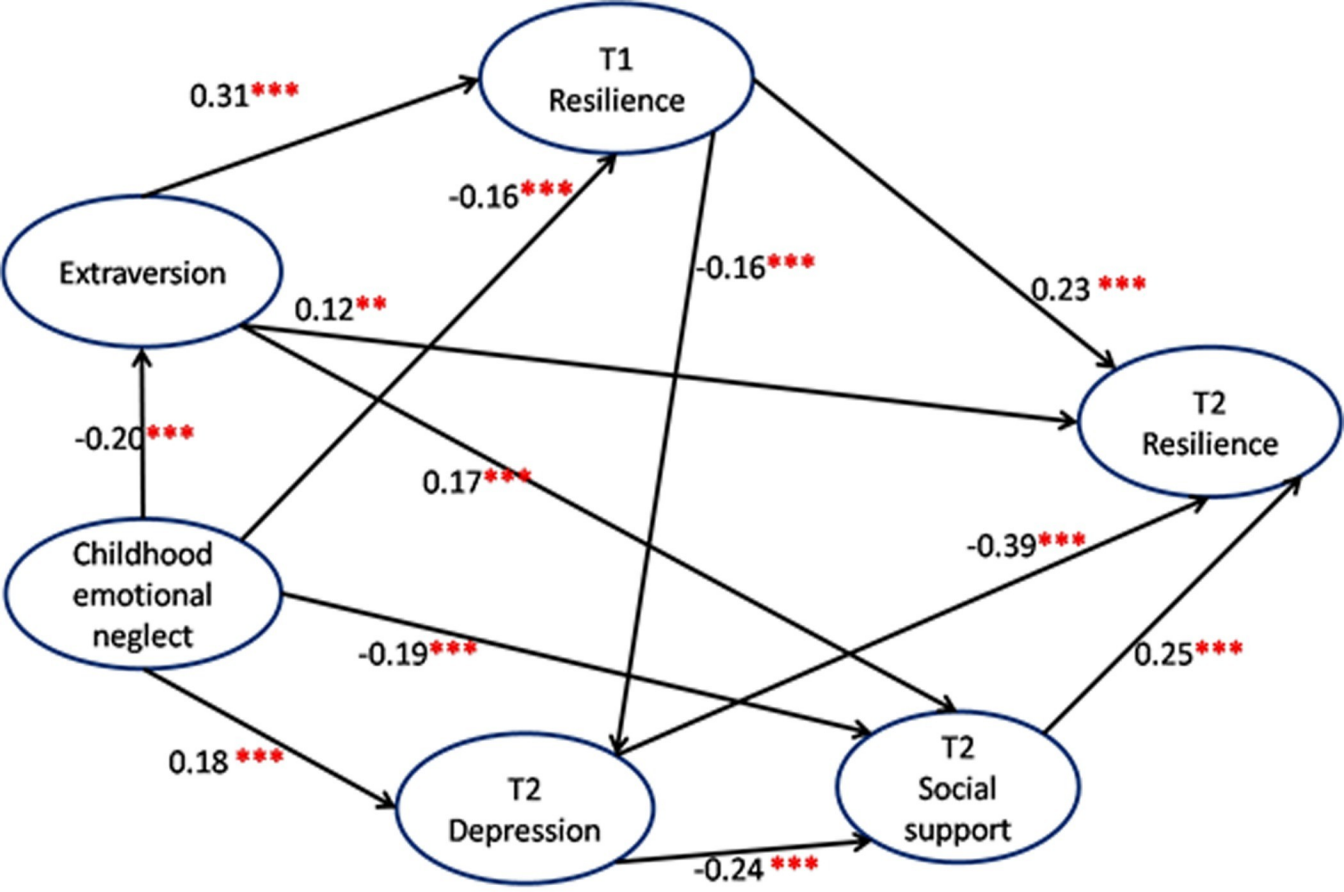
Structural equation model for T2 resilience. Note: *** P < 0.001. ** P < 0.01. T1 = Year 2016. T2 = Year 2018. Key information of model: CFI = 0.995, IFI = 0.996, NFI = 0.989, RFI = 0.944, RMSEA = 0.039, CMIN/DF = 1.65.

**Table 5:** Indirect effects and bootstrap analysis of models.

Model	Path	Effect value	95%CI		p
			Lower bounds	Upper bounds	
**T1 model**	Extraversion→T1 D	0.176	0.122	0.238	0.001
	T1 ASLEC→T1 D	-0.186	-0.256	-0.129	0.002
	T1 D →T1 D	-0.138	-0.207	-0.086	0.001
**T2 model**	EN→T2 R	-0.227	-0.294	-0.162	0.002
	Extraversion→T2 R	0.134	0.086	0.190	0.002
	T1 R→T2 R	0.071	0.032	0.106	0.002
	T2 D →T2 R	-0.06	-0.095	-0.035	0.001

Note: T1 = Year 2016. T2 = Year 2018. EN = Emotional neglect. R = Resilience. D = Depression. ASLEC = Adolescent Self-Rating Life-events Checklist.

Based on the regression results, we developed a hypothesis-driven (Hypothesis 1: long-term psychosocial vulnerabilities, i.e., early-life trauma and personality (i.e., extraversion) may have remote effects on early-adulthood resilience and Hypothesis 3: Current life-events, social support, and depression may play a short-term mediating role in the relationship between long-term psychosocial vulnerability and early-adulthood resilience) structural equation model with the variables of childhood EN, extraversion of personality, T1 resilience, T2 depression, social support, and resilience. Potential constructs between variables were based on the literature. Non-significant pathways were removed from the final model. The model fit test showed the fit index, CFI = 0.995, IFI = 0.996, NFI = 0.989, RFI = 0.944, RMSEA = 0.039, and CMIN/DF = 1.65, which indicated that the model fit was pretty good. Fig 2 shows that there were direct and positive effects of extraversion (0.12, p< 0.01) on T2 resilience, and indirect and negative effect of childhood EN (-0.227, p = 0.002) and a positive effect of extraversion (0.134, p = 0.002) on early-adulthood resilience, the corresponding 95% CI (-0.294, -0.162; 0.086–0.19) without 0, indicating a significant mediating effect between childhood EN, extraversion and T2 resilience. Moreover, Table 5 shows that there were direct and partial mediating positive effects of T1 resilience (direct effect = 0.23, p < 0.001; indirect effect = 0.071, p = 0.002), and a negative effect of T2 depression (direct effect = -0.39, p < 0.001; indirect effect = -0.06, p = 0.001) on T2 resilience, while T2 social support only had direct effect on T2 resilience (0.25, p <0.001). In which, childhood EN and T2 depression (strongest risk factor) had negative prediction on T2 resilience, while extroversion, T1 resilience and T2 social-support (strongest protector) had positive predictions on T2 resilience.

## Discussions

Based on the ecological system theory, the current study was among the first to suggest a longitudinal temporal process underlying the development of early-adulthood resilience using long-term and short-term psychosocial predictors. Conclusively and briefly, the findings showed that childhood EN and extraversion had a remote impact on early-adulthood resilience through T1 resilience, T2 depression and social-support, which suggest a temporal cumulative influence of longstanding childhood EN and extraversion on early-adulthood resilience through a medium- and short-term mediation of depression and social-support. The results support Bronfenbrenner’s ecological system theory model [8] regarding early-adulthood resilience in that, i.e., there are combined effects of long-term childhood-trauma and extraversion, medium- (two years before) and short-term (this year) depression and social-support on the development of early-adulthood resilience.

The current investigation showed that the resilience scores of the participants at T1 and T2 were identical (72.98 and 73.21, respectively), which were a little higher compared with previous results on resilience in Korean young adults (male: 63.88 ±15.33 vs. female: 55.60 ± 15.70) [55] and Chinese adolescents (64. 70 ± 18. 34) [56]. The findings showed that the resilience level of the current sample was relatively high, the reason might be that they were military students, whose resilience level might be guaranteed by less life-events or more social-support from group living.

Person correlation analysis revealed that early-life resilience was negatively correlated with EA, EN, PN, psychoticism and neuroticism of EPQ, depression and life-events at T1 and T2, and positively correlated with extraversion, and social support at T1 and T2. In other words, early-adulthood resilience might be influenced by long-term early-life trauma and extraversion, medium- and short-term life-events, social support, resilience, and depression.

In the regression and structural equation model, one unique early-life trauma variable included in both models was childhood EN, which indirectly predicted T1 and T2 early-adulthood resilience. Early-life trauma comprises of EA, PA, SA, EN, and PN [57]. EN is a type of abuse in which a parent withholds love or nurture from their child [58], which influence resilience in childhood [56] and adulthood [59]. The results of this study broadened our knowledge of early-adulthood resilience by demonstrating that childhood EN has a significant long-term predictive effect on early-adulthood resilience (Hypothesis 1: long-term psychosocial vulnerabilities, i.e., early-life trauma may have remote effects on early-adulthood resilience). This study is the first of its kind, expands our understanding of the fostering of early-adult resilience, and provides implications for future family and school education, i.e., childhood EN should be avoided in families, and individuals who experienced childhood EN should be intensively attended to, with compassionate meditation [60] or loving-kindness meditation [61] to erase the effect of EN trauma during childhood. As expected, in both regression and Amos equation models, extraversion had a positive and direct prediction on T1 and T2 resilience, as well as a positive and indirect prediction on resilience through the mediation of recent resilience, current depression and social-support. The results were quite consistent with those of previous studies on the role of extraversion in general resilience [17], i.e., extraversion acted as a longstanding psychological predictor (Hypothesis 1: long-term psychosocial vulnerabilities, i.e., personality (i.e., extraversion) may have remote effects on early-adulthood resilience). This study broadens the base of knowledge of early-adulthood resilience. Notably, extraversion predicted both T1 and T2 resilience directly and significantly, while childhood EN only predicted T2 resilience and none of the childhood trauma events predicted T1 resilience significantly. The results suggest a more stable prediction of extraversion on resilience compared with childhood trauma. In another words, endogenous long-term psychological vulnerability is a more stable predictor of early-adult resilience compared with exogenous long-term difficulties.

Importantly, regression and Amos equation analysis showed that the prediction of childhood EN and extraversion on early-adulthood resilience were partially but significantly mediated by recent and current depression, life-events and social-support. Specifically, current depressive levels had moderate direct and strongest negative predictions of early-adulthood resilience [7]. Resilience of two years ago, had a direct and positive prediction of current resilience. The current level of social-support, in contrast, had moderate direct and strongest positive prediction of early-adulthood resilience [39], and mediated the effect of childhood EN and extraversion, as well as past resilience and current depression on early-adulthood resilience, which suggested that social-support was an important protective mediator of resilience. The results suggest that in the collectivistic Chinese culture, support from family and society is important for fostering resilience during the early-adulthood period. The variable of life-events was previously reported to be negatively correlated with resilience [30], and was a risk factor for T1 resilience. Taken together, the results point to a cumulative impact of longstanding childhood EN and extraversion, recent resilience, and current life-events, depression and social-support on early-adulthood resilience (Hypotheses 2 and Hypotheses 3: recent and current life-events, social support, and depression may play a medium-term mediating role in the relationship between long-term psychosocial vulnerability and early-adulthood resilience), which helped to reveal the longitudinal temporal process underlying the development of early-adulthood resilience using long-term (childhood EN and extraversion), medium-term (resilience of two years ago), and short-term (current life-events, depression and social-support) predictors. To the best of our knowledge, this study is the first on this topic. Therefore, it is of great importance to identify people with a history of early-life trauma, and put more efforts into fostering resilience in early-adulthood through focusing on social-support enhancement, minimizing adverse life-events in the present, and reducing depression symptoms [7].

In the regression analysis, the current psychosocial-status (life-events, resilience, and social-support) accounted for 29.6% and 21.15% of the variance in T1 and T2 resilience, long-standing trauma and personality accounted for 14.4% and 15.8% of the variance in T1 and T2 resilience, while the recent psychosocial-status accounted for 7.35% of the variance in T2 resilience. In Amos model-test, the current psychosocial-status (life-events, resilience, and social-support) had the biggest pathway coefficients in both T1 and T2 models on resilience compared with recent psychosocial-status or stable longstanding psychosocial vulnerability. The results suggest that the current psychosocial-status have the strongest prediction on resilience. Thus, in fostering early-adulthood resilience, improving current psychosocial-status needs to be prioritized.

This study had several limitations: First, our study relied on retrospective reporting, and self-reported experiences of childhood trauma were not externally validated. However, memory of childhood trauma has been reported to be the most accurate [62]. Second, the gender ratio in this study was not identical (more males compared with females), which might result in a confounding effect of early-life trauma (e.g., SA) [63] due to unclassified gender effect, so we should be very cautious in generating the results. Third, the sample size of this study was not huge, which reframed the explanation power of the results. However, we calculated Cohen’s d for t-test, and evaluated the effect of regression according to Cohen’s guidelines, which indicated the effect size of this study. Four, due to the analysis methods, an alpha adjustment for familywise error or a power analysis were not carried out for regression analysis in the current study, which can not suggest a strong power in the significant paths. Finally, the current sample was 18 to 22 year olds medical university students, which could not fully represent the early-adulthood population (18–25 years old).In sum, based on the ecological system theory model, this study is among the first to observe the cumulative influence of longstanding childhood EN and extraversion on early-adulthood resilience through a medium- and short-term mediation of depression and social-support. The findings that early-life trauma and extroversion have a remote impact on early-adulthood resilience through recent and current life-events, depression and social-support suggest a temporal cumulative impact of longstanding early-life EN and extraversion on early-adulthood mental health. Our results have theoretical implications regarding early-adult resilience, i.e., early-life trauma does not hinder the fostering of early-adulthood resilience in a linear trend in medical university students. The findings also imply that extraversion acts as a long-term mediator, and later psychosocial variables such as life-events, depression and social-support are dynamic predictors.

## Supporting information

S1 Data (CSV)
